# Synthetic 3D printed tibial plateau with gradient material properties for biomechanical accuracy

**DOI:** 10.3389/fbioe.2025.1707380

**Published:** 2025-11-27

**Authors:** Damiano Coato, Gianmarco Dolino, Alice Berardo, Elisa Belluzzi, Assunta Pozzuoli, Pietro Ruggieri, Emanuele Luigi Carniel, Paolo Gargiulo

**Affiliations:** 1 Department of Engineering, Institute of Biomedical and Neural Engineering, Reykjavik University, Reykjavik, Iceland; 2 Centre for Mechanics of Biological Materials, University of Padova, Padua, Italy; 3 Department of Industrial Engineering, University of Padova, Padua, Italy; 4 Musculoskeletal Pathology and Oncology Laboratory, Department of Surgery, Oncology and Gastroenterology (DiSCOG), University of Padova, Padua, Italy; 5 Department of Orthopedics and Orthopedic Oncology, Department of Surgery, Oncology and Gastroenterology (DiSCOG), University-Hospital of Padova, Padua, Italy

**Keywords:** biomechanical properties, 3D printing, cartilage, mechanical testing, Polyjet

## Abstract

**Introduction:**

This study presents the design and fabrication of a synthetic 3D printed tibial plateau, complete with tibial cartilages, developed to replicate the mechanical behavior of its natural counterpart.

**Methods:**

Patient-specific anatomical data were used to design the model, which was fabricated using advanced PolyJet™ multi-material printing. Gradient material properties were integrated within the construct to reproduce the stiffness variations observed in native cartilage. Three different material mixes were developed and tested under indentation loading, and the optimal configuration (Mix 3) was selected based on its mechanical fidelity to biological tissue.

**Results:**

Mix 3 successfully reproduced the regional stiffness variations of native tibial cartilage. The instantaneous modulus (IM) of the synthetic cartilage closely matched that of the biological sample, with values of 3.19 ± 1.95 MPa vs. 3.31 ± 2.33 MPa in the lateral compartment and 3.71 ± 1.38 MPa vs. 3.72 ± 2.56 MPa in the medial compartment. Statistical analysis confirmed that most regional comparisons showed no significant differences (p > 0.05), supporting the strong mechanical agreement between synthetic and native cartilage.

**Conclusion:**

This study demonstrates the potential of Digital Anatomy materials produced with PolyJet™ technology as a viable method for 3D printing anatomically and mechanically accurate models of the human tibial plateau. Overall, this approach provides a reproducible and ethically sustainable alternative to biological specimens, with implications for preclinical testing, implant design optimization, and the advancement of high-fidelity surgical training models.

## 1 Introduction

The tibial plateau and its overlying articular cartilage are critical load-bearing structures that enable smooth joint motion, distribute mechanical forces, and contribute to knee stability. Their complex mechanical behavior, characterized by regional variations in stiffness, is essential to maintain healthy joint function and is often compromised in degenerative conditions such as osteoarthritis (Belluzzi et al., 2023; Aubonnet et al., 2023; Pettenuzzo et al., 2023).

Current models used to study knee biomechanics generally rely on cadaveric and animal specimens, computational simulations, or synthetic surrogates (Belluzzi et al., 2023). Each approach presents notable limitations. Cadaveric tissues, whereas anatomically accurate, exhibit high donor variability and degrade rapidly, making them unsuitable for standardized testing or long-term studies (Woo et al., 2006). Due to the difficulty in obtaining human cartilage, researchers frequent use animal tissues instead; however, significant variability still exists across species and anatomical sites (Belluzzi et al., 2023). Computational models, though increasingly sophisticated, often rely on oversimplified assumptions that fail to capture the nonlinear, anisotropic, and viscoelastic properties of cartilage (Mow et al., 1980; Kazemi et al., 2013; Cooper et al., 2019; Madeti et al., 2015). Synthetic models, on the other hand, frequently lack biomechanical fidelity and do not replicate the regional heterogeneity of cartilage structure (Belluzzi et al., 2023; Mandrycky et al., 2016; Vijayavenkataraman et al., 2018; Bhardwaj et al., 2015; Gu et al., 2023; Todros et al., 2022). These constraints limit reproducibility, standardization, and translational potential.

Three-dimensional (3D) printing has emerged as a powerful tool in clinical practice for the fabrication of patient-specific anatomical models used in surgical planning, professional training, device evaluation, and medical education (Soni et al., 2025). These models have shown to improve clinical outcomes by enhancing preoperative preparation, reducing operating room and imaging time, and increasing patient understanding (Stratasys, 2022a). However, they are often produced with materials that are not engineered to replicate the mechanical properties of biological tissues, limiting their effectiveness in applications and requiring realistic haptic feedback or load-bearing simulation (Bezek et al., 2022; Lee et al., 2020).

PolyJet™ 3D printing offers a promising solution. This technology enables the deposition of multiple acrylic-based photopolymers with micrometric-level precision using piezoelectric print heads and UV curing (Liu et al., 2020). It allows for the simultaneous jetting of elastomeric and rigid components, which can be combined in controlled ratios to create Digital Anatomy (DA) materials (Stratasys, 2025). These tunable materials are specifically designed to mimic the mechanical properties of biological tissues. This material versatility supports the fabrication of highly realistic, patient-specific models that replicate both the geometry and biomechanical behavior of human tissues (Palanisamy et al., 2022).

Recent advances in voxel-level and gradient-controlled 3D printing have improved the structural and mechanical fidelity of PolyJet™ -based models. By assigning material compositions at the voxel or sub-voxel scale, these methods enable precise control of local stiffness gradients and internal architecture (Guy et al., 2022; Saldívar et al., 2023; Bader et al., 2018). This spatially resolved deposition enhances reproducibility and allows the replication of zonal-dependent mechanical behavior observed in native tissues.

Tissue-mimicking 3D printed models provide a practical, ethical, and cost-effective alternative to cadaveric or animal specimens (Raeker-Jordan et al., 2022). They offer consistent, reproducible results by eliminating inter-sample variability (George et al., 2017). These models are customizable, durable, and reusable, making them particularly suited for surgical simulation, preoperative planning, and medical device testing, enhancing education and research without the limitations of biological tissue (Stratasys, 2022a; Brumpt et al., 2023).

Several studies have shown the potential of anatomical models produced using PolyJet™ technology and DA materials to replicate the behavior of biological tissues. These have been used to mimic the mechanical properties of various soft and hard tissues, including myocardium (Lee et al., 2020; Severseike et al., 2019), liver, subcutaneous tissue (Lee et al., 2020), vascular structures (Sommer et al., 2020; Sparks et al., 2021), and bone (Mustahsan et al., 2021; Forni et al., 2024a; Stratasys, 2022a). In knee joint applications, tendons and ligaments printed with bio-inspired infill patterns have exhibited tensile properties and range of motion comparable to those of native tissues (Grimaldo Ruiz et al., 2022). Similarly, knee joint soft tissue analogues incorporating fibers matrix architectures have withstood repeated flexion-extension cycles, successfully replicating the stress-strain behavior of real tissues (Ruiz and Dhaher, 2021). Despite these advances, a critical gap remains in the replication of load-bearing soft tissues such as articular cartilage. To date, simulations of knee cartilage using DA materials have been only marginally explored (Ciliberti et al., 2023). Most existing models fail to reproduce the complex structural organization, regional mechanical variability, and load-bearing functionality of the native tissue.

Building on these developments and addressing the gaps, the present study applies gradient-controlled Polyjet™ printing and Digital Anatomy materials to reproduce the patient-specific stiffness distribution of tibial cartilage, achieving both anatomical accuracy and mechanical tunability. The tibial plateau was chosen for its key load-bearing role in knee biomechanics, its clinical importance in cartilage degeneration, and the availability of a well-preserved specimen enabling accurate 3D reconstruction and validation against native tissue. Indeed, the final objective is to replicate the mechanical behavior of an individual, patient-specific tibial plateau. Due to the well-known high inter-subject variability in cartilage and subchondral tissue properties, including differences related to anatomy, age, and health status, our approach intentionally focuses on the intra-subject characteristics, thus mimicking the unique mechanical features of a specific individual, rather than to match population-level average behavior. By mapping the *in-situ* stiffness distribution across the human tibial surface and translating this data into a patient-specific DA material configuration, a synthetic model capable of reproducing the biomechanical complexity of the tibial cartilage has been developed. This approach provides a reproducible, scalable, and clinically relevant tool for orthopedic device testing, surgical planning, diagnostics, and biomechanical research. Beyond replicating healthy cartilage mechanics, the same workflow can be extended to simulate pathological conditions–such as focal lesions, degeneration, or post-surgical alterations–by integrating patient-specific imaging and mechanical data. This enables the development of personalized phantoms that reflect the progression or variability of joint diseases, enhancing fidelity and reproducibility in both experimental and clinical contexts.

## 2 Materials and methods

### 2.1 Sample collection and preparation

**FIGURE 1:**
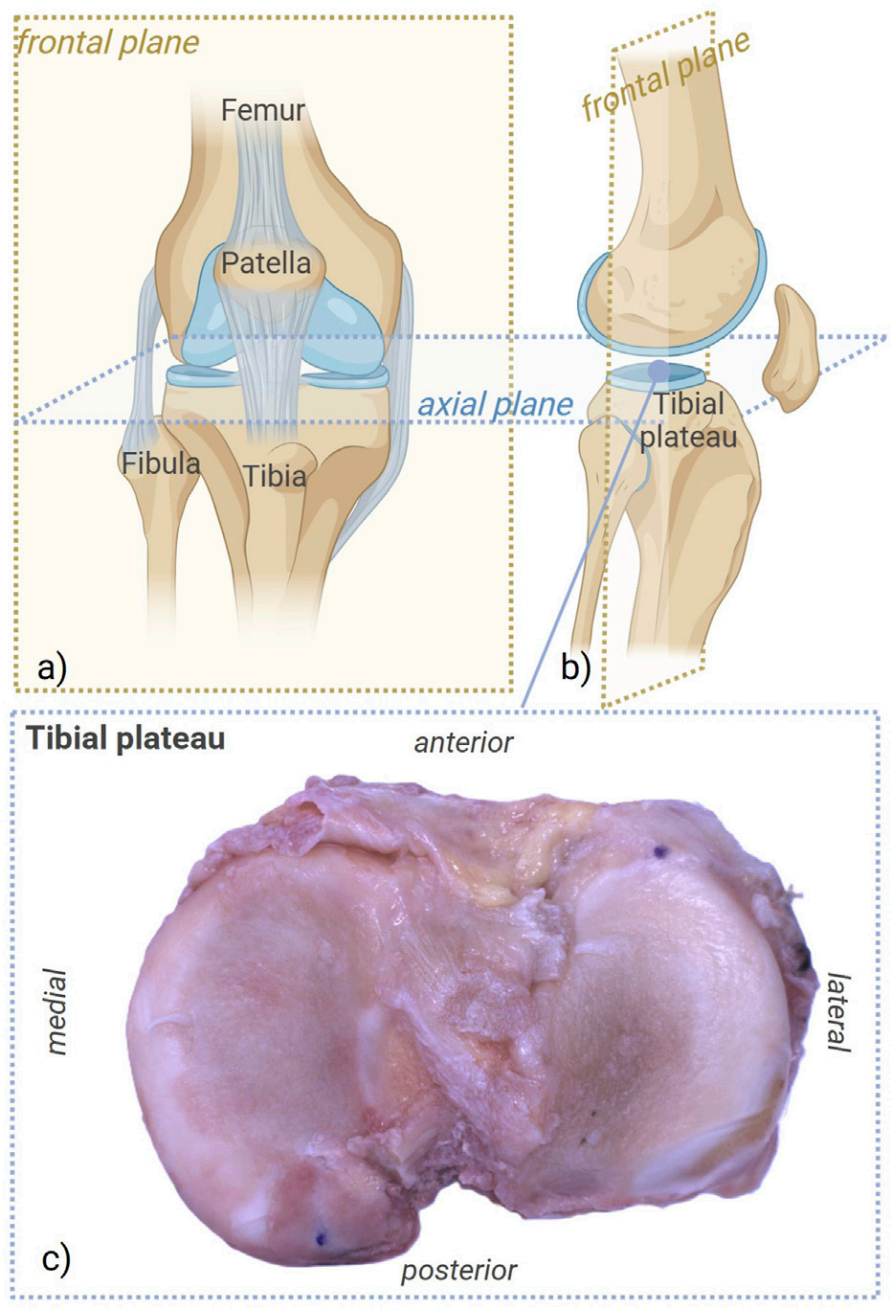
Anatomical references of the analyzed region, with details of the healthy tibial plateau, prior to mechanical testing. **(a,b)** Details of the frontal plane. **(c)** Zoom view on the biological tibial plateau. Created with BioRender.com.

A tibial plateau sample was provided by the Orthopedic Clinic of the University Hospital of Padova, obtained from a 80-year-old male donor with a body mass index of 26.7 $kg/m^{2}$ , who underwent right thigh amputation due to a leiomyosarcoma at the hip. The patient did not receive any neoadjuvant radiotherapy or chemotherapy prior to amputation. Given the anatomical distance from the tumor site, the tibial plateau was assumed to be free of pathological alterations and considered representative of healthy tissue, aside from age-related changes. The study was approved by the Local Ethical Committee of Padova (CESC Code: 5474/AO/22), and the patient gave the written informed consent to be included in the study. The sample consisted of a small part of the right proximal tibia and the overlying medial and lateral cartilages (Figure 1); no history of osteoarthritis was reported and no signs of age-related degeneration were observed. The tibial plateau was stored at −20° C immediately after amputation. It was then thawed overnight at 4° C immersed in a saline solution, and subsequently tested the following day.

### 2.2 Mechanical testing

Mechanical testing was conducted using a Mach-1 Model V500css test device (Biomomentum Inc., Canada) equipped with a 70 N multi-axial load cell, a spherical indenter of 0.5 mm radius to perform indentation (selected to minimise edge effects and excessive substrate influence, while ensuring a quite wide surface analysis), while a needle of 0.3 mm diameter and 12.5 mm length (BD PrecisionGlide™) to measure cartilage thickness.

Room temperature was controlled (25 °C) as well as humidity (75%). The indentation mechanical test was selected since it allows for localised, non-destructive characterisation of small, heterogeneous, and curved specimens such as tibial cartilage. Unlike uniaxial or biaxial tension/compression tests, these measurements included site-specific mechanical properties without requiring specimen excision, flattening, or special gripping conditions, which can alter the native tissue structure. Moreover, indentation closely replicates physiological loading conditions, where cartilage is typically loaded under confined or semi-confined compression.

Data from the mechanical tests performed on biological and synthetic samples were computed by means of the software Mach-1 Mapping Toolbox and Analysis (Version 4.1.0.19, Biomomentum Inc., Canada) combined with Matlab R2024b (Mathworks, United States).

#### 2.2.1 Mapping

**FIGURE 2:**
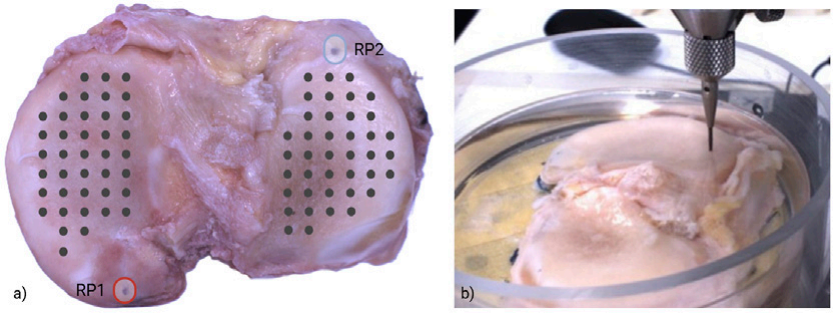
**(a)** Indentation map (black dots) on both medial and lateral tibial plateau; reference points (RP1 and RP2) were placed for image calibration, i.e., obtaining the coordinates of the measurement points. **(b)** Sample placement during indentation, immersed in saline solution. The indentation was repeated at each point reported in the indentation map.

The Mapping Toolbox software (Biomomentum Inc., Canada) was used to create an automated map of indentation points on the surface of the sample. A total of 80 points (40 on the medial region, 40 on the lateral region, Figure 2a) were created. Two reference points were used for image calibration, to define the local coordinates of each measurement point and to automate the acquisition procedure.

#### 2.2.2 Normal indentation procedure

To perform indentation on the tibial plateau while including surface variations and morphometric characteristics, a normal indentation was utilized. This function of the testing apparatus accurately detects both the local height and orientation of the surface at specified positions; then, it records the applied load while simultaneously coordinating the three linear stages at different velocities. By guiding the spherical indenter along a predefined displacement profile aligned with a virtual axis, normal to the sample surface, it calculates the normal force at each point of measure (Figure 2b).

During the normal indentation procedure, the biological sample was completely immersed in saline solution. The contact criterion with the sample surface was set to 0.1 N , whereas the indentation amplitude and the indentation velocity were 0.3 mm and 0.05 mm/s , respectively, similar to another study in literature (Seidenstuecker et al., 2019). See the Supplementary Material for more considerations on these adopted values. Due to both viscoelasticity and poroelastic (fluid-flow) behavior, the selected loading rate can be seen to be sufficiently slow to avoid inertial effects and excessive hydrodynamic pressurization that dominate at very high speeds, but at the same time sufficiently fast to capture the instantaneous material stiffness.

#### 2.2.3 Needle penetration procedure

After the indentation test, the spherical indenter was substituted with a needle to perform a second procedure designed to obtain the cartilage thickness. Specifically, the needle probe was advanced at a constant velocity until it penetrated the cartilage surface and reached the underlying cartilage-bone interface; the force limit was set to 7 N , a value high enough to indicate bone contact and below the critical load for needle instability, well within the load cell capacity. The vertical thickness was quantified as the distance between the point of initial load detection, which indicates the contact with the cartilage, and the point of sharp load increase, corresponding to the detection of bone tissue. The needle penetration procedure was performed using the same map as for indentation.

#### 2.2.4 Instantaneous modulus

The Instantaneous Modulus (IM) at each position was obtained by fitting the normal load-displacement curve with the elastic model for indentation according to Hayes et al., 1972:

$$IM=\frac{P}{H}\cdot \frac{1-v^{2}}{2ak\left(\frac{a}{h}v\right)}$$

where P = load, H = indentation depth, a = radius of the contact region, ν = Poisson’s ratio, k = correction factor dependent on a/h and ν , and h = sample thickness, previously measured. A Poisson’s ratio equal to 0.5 was adopted, since the instantaneous response of cartilage can be assumed to be incompressible in this configuration, due to the poroelastic effects and to the sample being saturated with saline solution during the entire procedure (Belluzzi et al., 2023). The software Mach-1 Analysis automatically selects the correction factor k as a function of the ratio a/h and the Poisson’s ratio, based on the tables from Hayes et al., 1972. In this case, the value ranges from 1.14 to 1.67.

### 2.3 3D model generation

**FIGURE 3:**
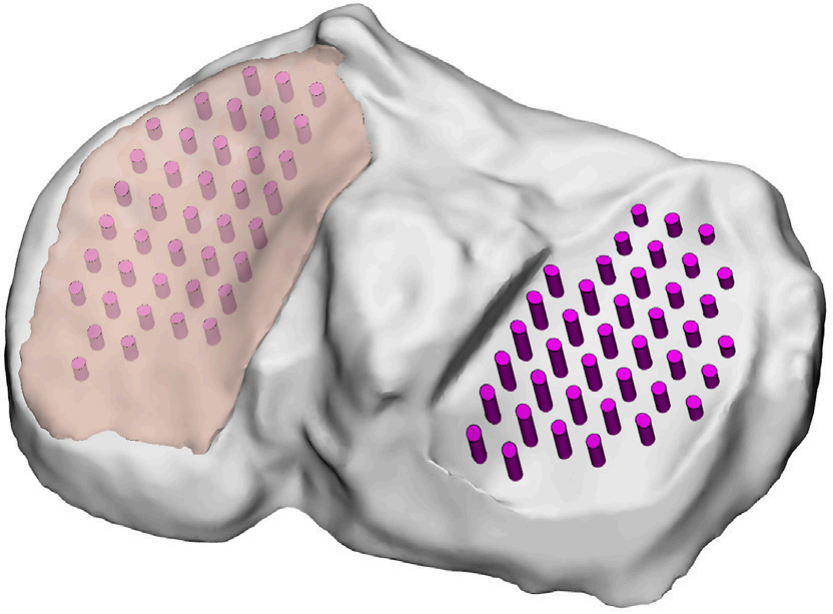
Experimental measurement points and cartilage layers on the reconstructed 3D model. Medial cartilage is represented as transparent, whereas lateral is omitted.

The biological sample was digitally reconstructed by means of the free software 3DF Zephyr (3D flow, 2022). A total of 50 pictures from multiple views of the sample were acquired and then imported into the software, which also performed triangulation to compute the spatial position of features and generated a dense point cloud. A polygonal surface mesh was then extracted to produce the initial 3D model. The mesh was exported as a point cloud format and processed in MeshLab (Visual Computing LabISTI - CNR, 2022) to clean, crop, and scale the model, assigning correct metric dimensions. Finally, Geomagic Design X (3D Systems, 2022) was used for surface refinement and solid model generation. To identify the areas of the tibial plateau covered by cartilage, the solid model was imported into SolidWorks (Systémes, 2022). Experimental measurement points from normal indentation and cartilage thickness mapping were projected onto the surface of the specimen. By assigning each projected point a vertical coordinate offset corresponding to the measured cartilage thickness, the lateral and medial cartilage-covered regions were identified, and obtained through interpolation of these points with parametric surfaces that recreated the interface between bone and cartilage. The remaining geometry, obtained by subtracting these regions from the global model, was considered to represent the underlying bone (Figure 3).

### 2.4 Additive manufacturing

#### 2.4.1 Material design

A preliminary selection of materials was conducted based on the results from a previous study (Forni et al., 2024b) to determine the most suitable combinations for this application. Tests evaluated both pure materials and 50%–50% (volume ratio) material blends under tensile and compressive loading conditions.

Since the focus of the study was on the characterization of cartilage properties through indentation tests, the effect of the underlying bone was considered negligible. However, to ensure mechanical stability and prevent substrate deformation during testing, a material approximately three orders of magnitude stiffer than the materials used to print the synthetic cartilages was selected for the bone structure. Specifically, VeroWhite™ was employed to reproduce the tibial plateau, as it has a compressive Young’s modulus of approximately 1540.00 MPa (Chen et al., 2017; Forni et al., 2024b). This value ensures that the substrate behaves as a rigid support during indentation, eliminating potential interference from its deformation. This approach follows standard practice in cartilage indentation testing, which commonly assumes a rigid substrate to eliminate compliance effects (Hayes et al., 1972; Argatov and Sabina, 2013). This material was chosen as the stiffest printable option available within the employed PolyJet™ system and has also been adopted in previous biomechanical phantom studies for its high dimensional accuracy and mechanical consistency, showing elastic properties comparable to those of biological bone tissue (Strand et al., 2024).

The first attempt (referred to as Mix 1) consisted of a gradient transition between two custom cartilage-like materials, previously designed in a separate study (Dolino et al., 2023) using GrabCAD Digital Anatomy Creator (DAC) 1.73 software (GrabCAD-Stratasys, United States). The first material was composed of 70% Agilus30™ (compression modulus = 1.13 MPa , Forni et al., 2024b), 15% BoneMatrix™ (compression modulus = 484 MPa , Forni et al., 2024b), and 15% GelMatrix™ (a gel-like support material), expressed as volume ratios. The second mix included 85% Agilus30™ and 15% BoneMatrix™, also expressed as volume ratios. The sample was printed with a material gradient transitioning from the first composition to the second, reproducing a progressive increase in stiffness from the inner to the external zones of the synthetic cartilage.

A second attempt (i.e., Mix 2) explored a new combination of materials to better match the properties of the biological sample. A mixture of Agilus30™, BoneMatrix™ and TissueMatrix™ (compression modulus approximately 0.30 MPa , Stratasys, 2022b) was used to replicate cartilage tissue. The mix was tuned in order to have a gradient of properties ranging from pure Agilus30™ to a 50%–50% BoneMatrix^TM^-TissueMatrix^TM^ blend (referred to as BMTM).

**FIGURE 4:**
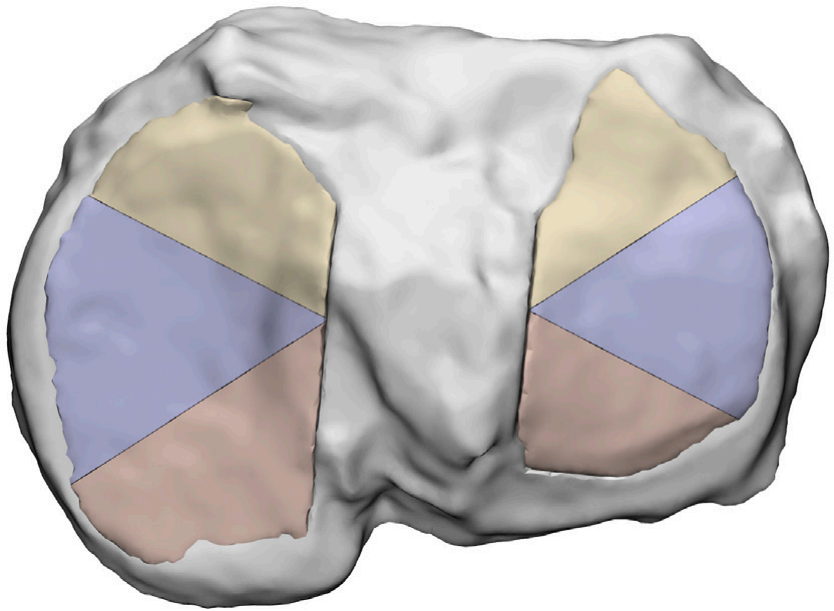
Sections of medial and lateral cartilages, after cut operation performed on 3-matic.

The DAC software includes a Noise Modulation tool that allows users to define gradual variations in properties by adding steps and adjusting how material volume percentages change across the model. The modulation process operates along the model’s longest axis, which is automatically detected by the software based on the model’s dimensions and thickness. However, the automatically defined longest axis was not considered appropriate for this study. To address this problem, each cartilage model was subdivided into three distinct sections at consistent angles. On 3-matic software, the models were cut along these sections (Figure 4) and the approach ensured that the DAC software’s built-in tool calculated the gradient axis in the desired orientation.

Finally, the third and last attempt (i.e., Mix 3) was an optimization of the second mix of materials (Agilus30™ and BMTM), with a different gradient distribution based on the result from the previous tests and the properties of the biological sample. Specifically, the modulus was reduced by incorporating pure Agilus30™ in the cartilage regions closest to the tibial condyles. In the areas of cartilage uncovered by the menisci, the modulus was slightly increased by enriching the gradient with more BoneMatrix™ and TissueMatrix™. In the outermost regions, only a 50:50 blend of BoneMatrix™ and TissueMatrix™ was used to achieve the highest modulus values. The detailed composition of Mix three is provided in the Supplementary Material for completeness.

#### 2.4.2 Printing process

Synthetic tibial plateaus (bone and cartilages) were printed using the J850 Digital Anatomy™ Printer (Stratasys, United States). Each model was manufactured with a matte surface finish. The printer was set at a precision of 27 μm (High Mix mode). Three sets of models with differently mixed materials were realized, as described in the previous section; three specimens for each mix to ensure reproducibility, for a total of nine printed tibial plateaus. After the process, all specimens underwent a manual cleaning process to remove the residual support material. All printed models underwent the same mechanical tests performed on the biological sample.

### 2.5 Statistical analysis

Experimental data are reported as mean ± standard deviation. Normality of the data was assessed using the Anderson–Darling test for each group, including medial and lateral tibial plateau regions of both biological and synthetic samples. When normality was not confirmed, non-parametric statistical methods were employed. For pairwise comparisons between two independent groups, the Mann–Whitney test was applied. In analyses of different sub-regions of the tibial plateau, data that met the normality assumption (based on the Anderson–Darling test) were analysed using two-sample unpaired t-tests; otherwise, the Mann–Whitney unpaired test was used. The Levene test was adopted to assess the variance dispersion between two samples. All statistical analyses were performed using Minitab Statistical Software (Version 21.4, Minitab LLC, State College, PA, United States, Minitab, 2025), with a significance level set at α = 0.05.

## 3 Results

### 3.1 Thickness measurements

**FIGURE 5:**
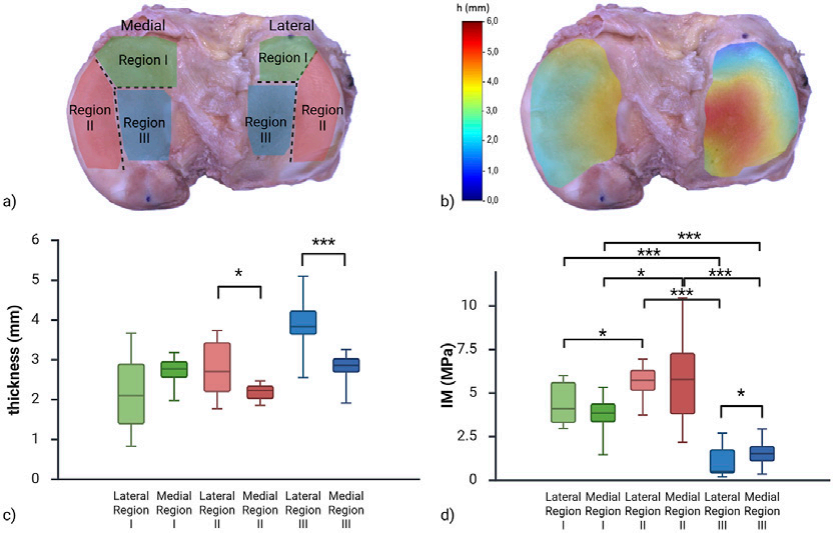
**(a)** Region subdivision for local properties evaluations. **(b)** Color map of thickness obtained from the needle probe measurements on the human cartilage. **(c)** Box plot reporting thickness distributions among lateral and medial regions. Significant statistical tests are reported (*p < 0.05, **p < 0.01, ***p < 0.001) **(d)** Box plot reporting IM distributions among lateral and medial regions. Created with BioRender.com.

The medial and lateral parts of the tibial plateau were subdivided into multiple regions (Figure 5a). Specifically, three regions were identified: anterior meniscus-covered (Region I), exterior-posterior meniscus-covered (Region II) and meniscus-uncovered (Region III), based on the results reported in Deneweth et al., 2013. The thickness color map of the biological sample is shown in Figure 5c. Articular cartilage thickness varied across regions of the tibial plateau depending on meniscal coverage, as highlighted in Figures 5c,d. Region III of both sides, corresponding to the meniscus-uncovered area, exhibited greater thickness compared to Regions I and II, which were covered by the meniscus. In particular, Region III was approximately 76% thicker than Region I (anterior portion of the meniscus-covered area) in the lateral compartment, and 28% thicker than Region II in the medial compartment. In contrast, cartilage in the meniscus-covered regions (Regions I and II) showed similar thickness between regions and compartments (Table 1). Statistical analysis between compartments of the same regions resulted in a statistical difference between Regions II (lateral vs. medial p = 0.02) and Region III (p < 0.0001).

**TABLE 1:** Cartilage thickness expressed in mm , average ± SD for each region.

Compartment	Average	Region I	Region II	Region III
Lateral	3.15 ± 1.00	2.18 ± 0.96	2.79 ± 0.67	3.84 ± 0.64
Medial	2.55 ± 0.41	2.71 ± 0.36	2.20 ± 0.18	2.82 ± 0.33

### 3.2 Indentation test on the biological sample

The identification of the instantaneous modulus was performed including the effect of the thickness variation along the sample, obtained through the needle penetration procedure. On average, cartilage of the biological tibial plateau (referred to as TPB) displayed an IM of 3.53 ± 2.45 MPa , with a clear variation in both compartments: the lowest stiffness concentrated centrally, corresponding to the region not covered by the meniscus and progressively increasing toward the periphery (Figure 5b). In the medial compartment, the IM ranged from 0.35 MPa in the central region to value of 10.46 MPa in the peripheral areas, particularly toward the external edge. Similarly, the lateral compartment exhibited an IM range between 0.19 MPa and 6.95 MPa . The central region showed the lowest values, with stiffness increasing toward the anterior and posterior edges.

Regarding the comparison between the regions, focusing on lateral versus medial ones, the non-parametric Mann-Whitney unpaired test reported a significant difference (p = 0.041) only for Region III. When comparing results between regions of the same zone (i.e., lateral or medial), significant differences were reported for all combinations, specifically, for lateral: Region I versus II (unpaired t-test p = 0.024), Region I versus III (unpaired Mann-Whitney p < 0.0001) and Region II versus III (unpaired Mann-Whitney p < 0.0001); for medial: Region I versus II (unpaired t-test p = 0.011), Region I versus III (unpaired t-test p < 0.0001) and Region II versus III (unpaired t-test p < 0.0001).

### 3.3 Comparison with synthetic samples (Mix 1, Mix 2)

**FIGURE 6:**
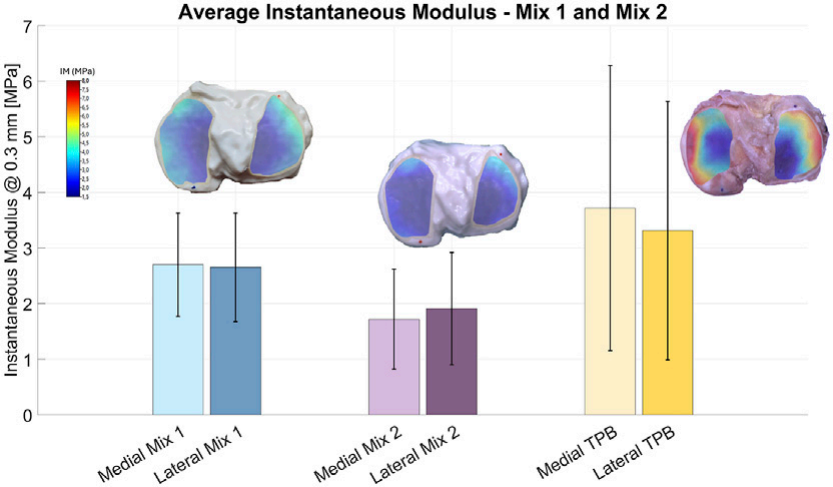
Average instantaneous moduli with standard deviations for synthetic tibial cartilage printed using Mix 1 (light and dark blue) and Mix 2 (light and dark purple); the right-most columns (light and dark yellow) show the corresponding results for the biological cartilage sample (TPB).

The instantaneous modulus at 0.3 mm indentation depth was measured for synthetic tibial cartilage produced with two different material formulations (Mix 1 and Mix 2) and compared with native biological cartilage (Figure 6).

Samples printed with Mix 1 exhibited average IM in the same order of magnitude of the biological one, although none of the samples reached IM values close to TPB, ranging from 2.55 MPa to 2.92 MPa . In the same way, Mix 2 samples showed even lower average moduli, ranging from 1.68 MPa to 1.88 MPa , with all samples presenting comparable stiffness and variability. Both solutions are statistically different from the biological sample (TPB), even when comparing the same region. In details, for Mix 1 all unpaired Mann-Whitney p < 0.05, for Mix 2 only lateral Regions I unpaired t-test p = 0.056 and lateral Regions III unpaired t-test p = 0.056, all the others unpaired Mann-Whitney p < 0.05.

Overall, these results indicate that both Mix 1 and Mix 2 not sufficiently mimic the IM of native cartilage, nor do fully replicate the mechanical behavior and variability observed in the biological tissue.

### 3.4 Design of the optimized solution (Mix 3)

**FIGURE 7:**
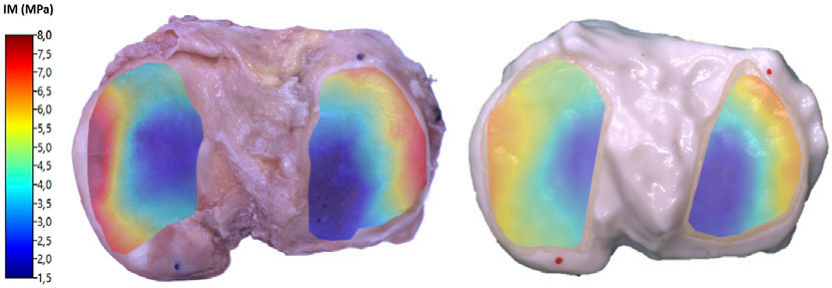
Color map of the instantaneous modulus calculated on the cartilage surfaces of TPB (left) and printed Tibial Plate #7, Mix 3 (right).

Although neither Mix 1 nor Mix 2 fully met the expectations in replicating the properties of TPB, the materials used in the latter combination were still considered the most suitable for the purpose. The base materials, Agilus30™ and the BoneMatrix™ - TissueMatrix™ (BMTM) blend, offered the potential to replicate the mechanical properties of native cartilage. However, the initial gradient configuration was only partially successful: whereas the overall behavior fell within the desired range, the extreme low and high modulus values were not sufficiently accurate (Figure 7). To address this issue and develop Mix 3, the same materials as in Mix two were used, but with an optimized gradient distribution as described in Section 2.4.1. Average IM values with standard deviations and the comparison with the biological sample are reported in Table 2.

**TABLE 2:** Instantaneous Modulus expressed in MPa , average ± SD for each region.

Sample	Average	Region I	Region II	Region III
TPB^*^ - lateral	3.31 ± 2.33	4.41 ± 1.24	5.64 ± 0.94	1.02 ± 0.87
TPB^*^ - medial	3.72 ± 2.56	3.74 ± 1.15	5.83 ± 2.52	1.60 ± 0.74
Mix 3 - lateral	3.19 ± 1.95	4.75 ± 1.03	4.96 ± 0.74	1.25 ± 0.43
Mix 3 - medial	3.71 ± 1.38	3.77 ± 0.51	4.68 ± 1.14	2.33 ± 0.73

^*^TPB, tibial plate biological.

**FIGURE 8:**
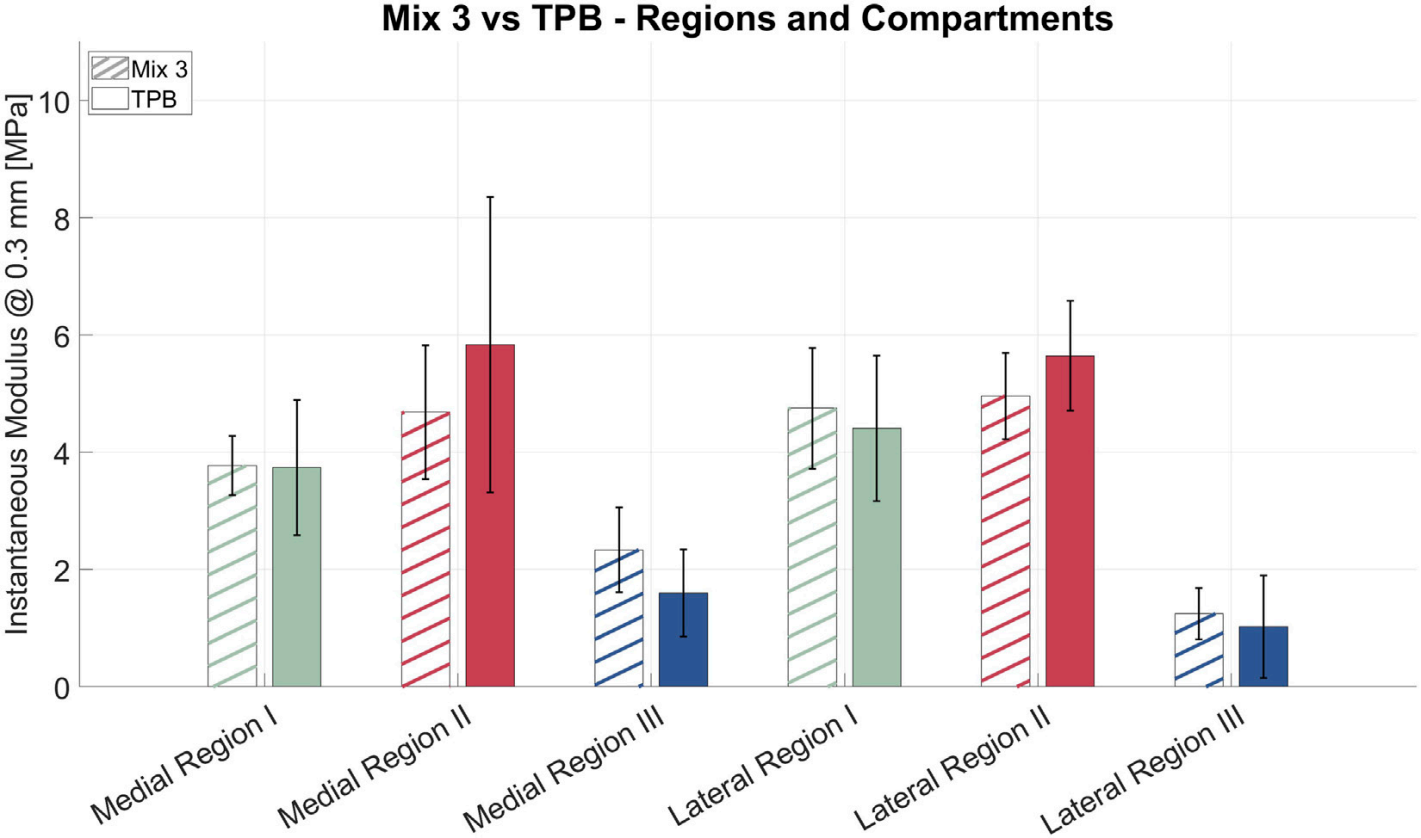
Zonal average IM with standard deviation of synthetic tibial cartilages printed using Mix 3 (with lines) and biological sample (full color). *p < 0.05, **p < 0.01.

Measurements are reported in Figure 8 for both the medial and lateral compartments, further subdivided by anatomical regions as described before. Most regional comparisons were not statistically significant (p > 0.05), except for lateral Region II (p = 0.042), and medial Region III (p = 0.002). Levene’s test confirmed equal variances (p = 0.91), supporting the validity of the comparison.

## 4 Discussion

### 4.1 Thickness distribution

Spatially distributed point measurements were performed across the entire cartilage surface to capture local variations in its thickness from one healthy tibial plateau. The presented findings confirmed the strong thickness variability across regions as well as between compartments (with the lateral being thicker than the medial one). The results are consistent with previous studies that reported thinner articular cartilage in meniscus-covered regions compared to uncovered areas (Seidenstuecker et al., 2019; Coleman et al., 2013). Indeed, Coleman et al., 2013 reported tibial plateau cartilage thickness similar to the obtained values (thus within the reported standard deviations in males), even if measured with a different strategy (e.g., from MRI). Moreover, the results align with Thambyah et al., 2006, who found significantly reduced cartilage thickness in meniscus-covered regions of the tibial plateau. Similar to their Group I and II (meniscus-uncovered areas of lateral and medial plateaus), Region III in the present study (uncovered area) showed the highest cartilage thickness. Although the absolute thickness values reported were slightly different due to common inter-subject variability, the relative differences between the covered and uncovered areas were comparable, suggesting a consistent biomechanical influence of the meniscal coverage on cartilage morphology.

### 4.2 Instantaneous modulus: biological

This study quantified the instantaneous modulus (IM) of cartilage at 80 sites across one human tibial plateau and nine 3D printed synthetic tibial plateaus aiming to mimic their biological counterpart. Concerning the biological testing, results confirmed that tibial articular cartilage exhibits non-uniform, region-specific mechanical properties, as evinced by Seidenstuecker et al., 2019; Deneweth et al., 2013.

From the statistical analysis, significant differences in IM were observed when averaged across the three respective regions of the medial and lateral plateaus. In both compartments, Region III (meniscus-uncovered) consistently showed the lowest average IM (see Table 2). In contrast, Region II (exterior and posterior meniscus-covered area) was significantly stiffer (p < 0.05) than both Regions I (anterior portion of the meniscus-covered area) and III (meniscus-uncovered area). These results suggest that the mechanical variability of tibial articular cartilage can be effectively characterized by three distinct regions: meniscus-uncovered, anterior meniscus-covered, and exterior-posterior meniscus-covered, listed in order of increasing stiffness. This supports previous findings (e.g., Deneweth et al., 2013) that reported significant variation not only between covered and uncovered regions, but also within the meniscus-covered area itself. Although this analysis was limited to a single human tibial plateau, comparison with previous studies suggests that the regional pattern of IM reflects a generalizable trend rather than subject-specific variability (Seidenstuecker et al., 2019; Deneweth et al., 2013). Moreover, no significant differences were reported between lateral and medial IM, in line with Seidenstuecker et al., 2019, where authors adopted a similar experimental protocol. Indeed, they reported an average IM of 3.43 ± 0.36 MPa overall. Specifically, they found an average IM of 4.94 ± 0.45 MPa in the meniscus-covered region and 1.79 ± 0.34 MPa in the meniscus-uncovered region. Additionally, the average modulus was 3.17 ± 0.47 MPa in the lateral compartment and 3.66 ± 0.55 MPa in the medial compartment. This strongly aligns with the presented results, reported in Table 2. A previous study by Thambyah et al., 2006 reported values ranging from 2.13 ± 0.74 MPa in the lateral, meniscus-uncovered region to 5.13 ± 1.91 MPa in the medial, meniscus-covered region, depending on anatomical location. These measurements were obtained using a load-controlled protocol, whereas the present study employed a displacement-controlled approach.

### 4.3 Instantaneous modulus: synthetic

The 3D printed model incorporated a designed gradient of material properties aimed at replicating the spatial heterogeneity of native tissue, as highlighted before. The same indentation point map and protocol were used for both the biological cartilage and the 3D printed ones, allowing for a direct comparison between native tissue and the synthetic model. The indentation results confirmed that the printed models obtained through Mix 3 successfully reproduced the regional stiffness variations. Despite minor deviations at specific locations - resulting from operator’s selection of gradient distribution - the printed samples mimicked the overall trend and spatial heterogeneity of the biological cartilage with high fidelity (Table 2). In detail, Mix 3 showed an IM close to that of native cartilage, particularly in Region I of both the medial and lateral compartments. In contrast, lower stiffness was observed in Region II compared to TPB, although the statistical tests were not significant for medial Region II (synthetic vs. biological); statistical tests were still significant (p = 0.042) for lateral Region II and for medial Region III (p = 0.002). Thus, since almost all comparisons between synthetic and biological regions yielded no statistically significant differences, it can be stated that the synthetic distribution pattern closely resembles that of the biological sample. This outcome underscores the potential of Digital Anatomy Materials and PolyJet™ additive manufacturing techniques to create synthetic models that not only replicate anatomical geometry but also approximate site-specific native mechanical properties.

In the existing literature, innovative strategies have begun to emerge for 3D printing heterogeneous knee tissues, notably the meniscus. Du et al., 2023 presented how 3D printing can be employed to recreate the meniscus inherent structural heterogeneity and anisotropy through tailored biomaterial constructs and printing architectures. This highlights the potential of additive manufacturing to faithfully replicate the complex fibrous organization of fibrocartilaginous tissues. Beyond this, other studies have made strides toward engineering anisotropic soft tissues using 3D printing. For instance, researchers have developed gradient-structured cartilage scaffolds that support heterogeneous chondrogenesis by mimicking native depth-dependent architecture, achieving mechanical anisotropy via variations in pore structure and bioink composition (Sun et al., 2021). Such advances show promise for regenerating layered, anisotropic cartilage tissues.

Nevertheless, when it comes to synthetic cartilage phantoms–designed to replicate mechanical behavior rather than bioactivity–there is still a significant absence of examples in the scientific literature. Indeed, to the authors’ knowledge, this is the first study to report the development of a tibial plateau phantom with such site-specific and mechanically heterogeneous characteristics. Addressing this gap by developing anisotropic synthetic cartilage phantoms would therefore represent a critical step toward more realistic biomechanical models and translational applications in both research and clinical practice. Future studies will aim to extend this approach to the entire knee joint, with phantoms designed to reproduce the distinct anatomical and mechanical features of the different constituent tissues.

### 4.4 Limitations and future directions

When interpreting the results of this study, some limitations should be acknowledged. First, the control analysis was conducted on a single human tibial plateau. Although this was considered acceptable given the main goal of evaluating 3D printing for patient-specific cartilage models, future studies should include a larger cohort to better assess robustness and reproducibility. Second, cartilage mechanics were characterised using a single parameter, the instantaneous modulus, which simplifies the tissue’s complex, nonlinear behavior (Belluzzi et al., 2023). However, this choice allowed direct comparison with previous studies and enabled the development of patient-specific phantoms with inhomogeneous properties that can be reproducibly manufactured and tested while preserving key mechanical contrasts.

In addition, at some measurement points, the selected indenter diameter slightly exceeded the dimensional constraints suggested by ISO 14577-1:2015, defined for indentation on metallic materials. The choice of a smaller indenter could have further reduced potential substrate effects. Third, the tibial plateau model was derived from stereophotogrammetry rather than high-quality CT, which may affect the accuracy and generalizability of the printed replicas. However, needle penetration measurements provided precise cartilage thickness data, thereby reducing discrepancies between the synthetic and the biological geometries at least in the measured points. Moreover, the results obtained from synthetic models were consistent with those of the biological sample and prior literature (Seidenstuecker et al., 2019). Finally, the materials employed were synthetic polymers that do not replicate cartilage composition, water content, or full mechanics. However, the purpose of this study was not to develop a biologically accurate substitute, but rather to evaluate whether 3D printing can reproduce key mechanical properties for patient-specific models in applications where only certain features are needed, such as surgical training, biomechanical testing, and implant evaluation.

Future studies should expand this work by incorporating a larger cohort of tibial plateau specimens, including pathological samples (e.g., osteoarthritic cartilage), to assess inter-subject variability and validate the method for diseased tissue. Higher-resolution imaging techniques, such as CT or MRI-based segmentation, will be explored to enhance anatomical fidelity. Mechanical characterization will also be extended beyond the instantaneous modulus to capture the viscoelastic response of cartilage through stress-relaxation or dynamic testing.

## 5 Conclusion

This study quantitatively validated the capability of Digital Anatomy materials and PolyJet™ 3D printing technology to replicate the patient-specific geometry and zonal mechanical behavior of human tibial cartilage. Through the integration of spatially graded material compositions, the optimized formulation (Mix 3) achieved matching values in both lateral and medial compartments. The statistical analysis showed no significant differences for most regional comparisons, confirming the ability of the printed constructs to reproduce the site-dependent mechanical response of biological tissue. These results demonstrate that controlled material gradients can effectively mimic the load-bearing properties and mechanical anisotropy of tibial cartilage, establishing a reproducible and tunable framework for patient-specific phantom fabrication. While biochemical and hydration-dependent aspects of cartilage remain beyond the scope of the current model, the achieved mechanical fidelity highlights the potential of this method for preclinical testing, implant optimization, and surgical simulation. Overall, the study provides quantitative evidence that multi-material 3D printing can bridge the gap between biological realism and experimental reproducibility in soft tissue modeling.

## Data Availability

The raw data supporting the conclusions of this article will be made available by the authors, without undue reservation.
