# Supplementary material for: Synthetic 3D printed tibial plateau with gradient material properties for biomechanical accuracy
Source: Front Bioeng Biotechnol. 2025 Nov 27;13:1707380. doi: 10.3389/fbioe.2025.1707380 (PMC12695809; doi:10.3389/fbioe.2025.1707380)
Supplement: Supplementary file 1 [file Presentation1.pdf]

Supplementary Material

1 SUPPLEMENTARY FIGURES AND TABLES

This supplementary material includes additional figures and tables supporting the gradient design, indentation tests, and material composition of the synthetic tibial plateaus.

1.1 Figures

1.1.1 Gradient Design

For the gradient design, each cartilage model was subdivided into three sections (Figure S1) to orient the DAC Noise Modulation tool (Figure S2) along the desired axis, allowing the correct assignment of the materials.

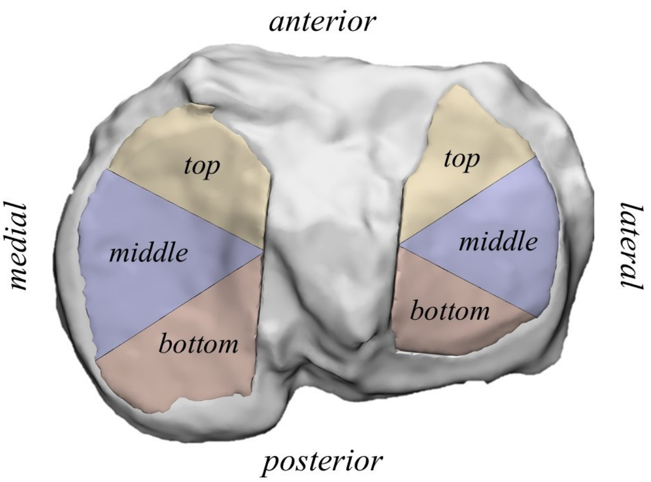

Figure S1: Sections and subdivision of medial and lateral cartilages, after cut operation performed on 3-matic.

1.1.2 Indentation Test

With reference to the general geometric constraints reported in ISO 14577-1:2015 for instrumented indentation testing, it is recommended that the indenter diameter does not exceed one-third of the sample thickness and that the indentation depth remains below one-tenth of the sample thickness. This standard was developed for metallic materials, but we can state that by increasing the indenter radius, this enlarges the contact area and the sampling volume, but also increases the risk of substrate influence when the cartilage layer is thin. For internal verification of potential effects related to the indenter diameter (worse case), we tested additional synthetic tibial plateaus using a 2 mm diameter indenter (Figure S3). However, no significant differences were observed (p-values: lateral compartment 1 mm vs 2 mm = 0.99; medial compartment 1 mm vs 2 mm = 0.40).

In addition, we also compared the obtained results by assuming an indentation depth of 0.2 mm instead of 0.3 mm. The following table reported the obtained results for the IM in both cases. Non-parametric

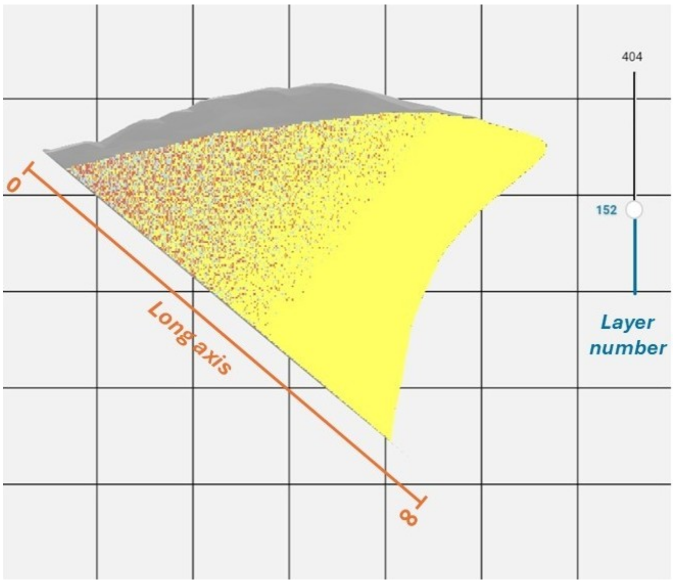

Figure S2: Graphical representation of the voxel-controlled Noise Modulation tool used to define gradient axes on GrabCAD Pro.

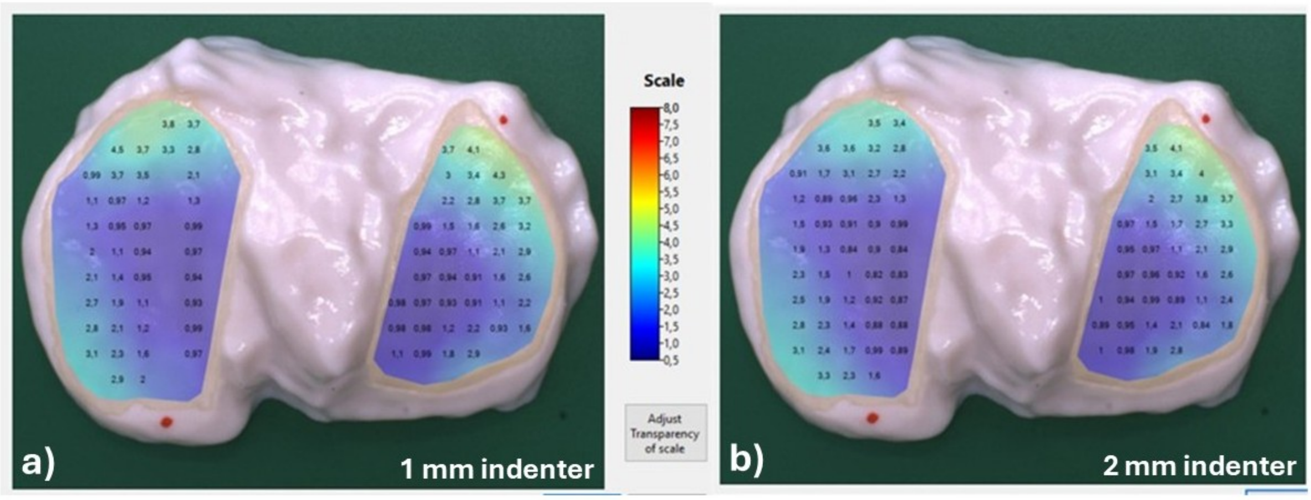

Figure S3: Color maps of the instantaneous modulus on the cartilage surfaces of the synthetic tibial plateau printed with Mix 2, obtained from indentation tests using a 1 mm indenter diameter (a) and a 2 mm indenter diameter (b).

comparisons between paired datasets were performed using the 1-Sample Sign test, to verify whether the median of the differences significantly deviated from zero. The p-value (0.727) indicated no significant difference between the two datasets, confirming that the obtained IM values were not significantly affected by the here chosen indentation depth.

Table S1: Instantaneous modulus (IM) computed at indentation depths of 0.20 mm and 0.30 mm, and corresponding difference values. NaN indicates measurements not analyzed due to excessive sample slope or unclear load–displacement curve.

IM @0.30 mm (MPa)	IM @0.20 mm (MPa)	Difference (MPa)
5.4834	5.6665	−0.1831
5.9434	5.9104	0.0330
5.9986	5.9914	0.0072
3.3169	3.4181	−0.1012
3.3330	3.3868	−0.0538
4.4141	4.4554	−0.0413
5.3878	5.5084	−0.1206
2.9683	3.0171	−0.0488
3.8171	3.8811	−0.0640
3.7453	3.8484	−0.1031
5.9305	5.9856	−0.0551
2.4094	2.4030	0.0064
1.6065	1.5933	0.0132
2.1362	2.1535	−0.0173
4.7851	4.8181	−0.0330
5.7266	5.7534	−0.0268
6.6618	6.7278	−0.0660
0.9862	0.9692	0.0170
0.4394	0.3575	0.0819
0.4480	0.3660	0.0820
NaN	NaN	
5.8950	5.9614	−0.0664
6.9473	7.1233	−0.1760
NaN	NaN	
NaN	NaN	
0.2495	0.2465	0.0030
NaN	NaN	
5.2196	5.2645	−0.0449
6.6726	7.1003	−0.4277
0.5080	0.4966	0.0114
NaN	NaN	
0.4998	0.3318	0.1680
1.8710	1.8385	0.0325
5.1154	5.4447	−0.3293
0.1900	0.1454	0.0446
0.2855	0.2617	0.0238
0.5588	0.5517	0.0071
2.7067	2.7250	−0.0183
NaN	NaN	

IM @0.30 mm (MPa)	IM @0.20 mm (MPa)	Difference (MPa)
0.4136	0.4074	0.0062
1.4665	1.4187	0.0478
3.6038	3.6277	−0.0239
4.1217	4.1758	−0.0541
4.3201	4.3172	0.0029
5.3274	4.5619	0.7655
3.4495	3.3220	0.1275
4.5116	4.5612	−0.0496
7.1027	7.1550	−0.0523
7.7739	7.7026	0.0713
3.1115	1.6633	1.4482
1.1468	1.0305	0.1163
1.6012	1.5335	0.0677
7.8261	7.9954	−0.1693
3.1722	3.3262	−0.1540
1.7148	1.5050	0.2098
0.6396	0.6406	−0.0010
0.3510	0.3519	−0.0009
10.4617	10.0549	0.4068
2.1776	2.1528	0.0248
1.8031	1.8492	−0.0461
1.3732	1.3562	0.0170
1.1398	1.1415	−0.0017
10.4541	10.0870	0.3671
4.0146	4.0556	−0.0410
2.3070	2.2411	0.0659
1.0571	1.0423	0.0148
0.9932	0.9730	0.0202
5.5846	5.5319	0.0527
4.1122	4.0391	0.0731
2.6271	2.6023	0.0248
1.4631	1.4406	0.0225
1.6969	1.6394	0.0575
7.0884	7.5024	−0.4140
3.2439	2.5183	0.7256
3.1519	3.0931	0.0588
2.7241	2.7638	−0.0397
2.9440	2.9535	−0.0095
5.9880	5.8952	0.0928
4.9008	5.0440	−0.1432
6.2633	7.3225	−1.0592

1.2 Tables

The specific compositions used in the synthetic samples printed with Mix 3 are reported below. All percentages refer to volume ratios.

Table S2. Summary of material composition of Mix 1, Mix and Mix3

Mix #	Material 1	Material 2	Material 3
1	Agilus30	BoneMatrix	GelMatrix
2	Agilus30	BoneMatrix	TissueMatrix
3	Agilus30	BoneMatrix	TissueMatrix

Table S3. Medial Cartilage - Bottom Compartment

Step (mm)	Agilus30 (%)	TissueMatrix (%)	BoneMatrix (%)
0	0	50	50
10	0	50	50
14	40	30	30
20	50	25	25
∞	100	0	0

Table S4. Medial Cartilage - Medium Compartment

Step (mm)	Agilus30 (%)	TissueMatrix (%)	BoneMatrix (%)
0	0	50	50
7	0	50	50
9	10	45	45
20	80	10	10
∞	100	0	0

Table S5. Medial Cartilage - Top Compartment

Step (mm)	Agilus30 (%)	TissueMatrix (%)	BoneMatrix (%)
0	0	50	50
9	20	40	40
12	100	0	0
∞	100	0	0

Table S6. Lateral Cartilage - Bottom Compartment

Step (mm)	Agilus30 (%)	TissueMatrix (%)	BoneMatrix (%)
0	0	50	50
8	50	25	25
13	100	0	0
∞	100	0	0

Table S7. Lateral Cartilage - Medium Compartment

Step (mm)	Agilus30 (%)	TissueMatrix (%)	BoneMatrix (%)
0	0	50	50
1	0	50	50
10	20	40	40
13	100	0	0
∞	100	0	0

Table S8. Lateral Cartilage - Top Compartment

Step (mm)	Agilus30 (%)	TissueMatrix (%)	BoneMatrix (%)
0	0	50	50
7.5	0	50	50
14	20	40	40
22	100	0	0
∞	100	0	0
